# Associations of systemic, serum lipid and lipoprotein metabolic pathway gene variations with polypoidal choroidal vasculopathy in China

**DOI:** 10.1371/journal.pone.0226763

**Published:** 2019-12-26

**Authors:** Ningda Xu, Hui Xu, Mingwei Zhao, Yongsheng Xu, Lvzhen Huang

**Affiliations:** Department of Ophthalmology, Peking University People’s Hospital Eye Diseases and Optometry Institute, Beijing Key Laboratory of Diagnosis and Therapy of Retinal and Choroid Diseases, College of Optometry, Peking University Health Science Center; Beijing,China; University of Miami, UNITED STATES

## Abstract

**Background:**

To investigate the association of systemic, serum lipids and genetic variants in the high-density lipoprotein (HDL) metabolic pathway with polypoidal choroidal vasculopathy (PCV) in China.

**Methods:**

The case-control study was included 150 controls and 66 cases with PCV. Serum levels of total cholesterol (TC), low-density lipoprotein (LDL), HDL, triglycerides (TG), apolipoprotein A1 (APOA1), apolipoprotein B (APOB) together with systemic risk factors including gender, hyperlipidemia, diabetes mellitus (DM), hypertension, coronary artery disease (CAD) and asthma were identified. All subjects were genotyped for four single nucleotide polymorphisms (SNPs) from three genes in the HDL metabolic pathway: rs10468017 of hepatic lipase (LIPC), rs12678919 of lipoprotein lipase (LPL), rs3764261 and rs173539 of cholesterol ester transfer protein (CETP) with matrix-assisted laser desorption/ionization time-of-flight mass spectrometry (MALDI-TOF-MS). Student’s t-tests, chi-square tests, anova and logistic regression were used to evaluate associations.

**Results:**

Hyperlipidemia was a risk factor (odds ratio (OR) = 1.19, P = 0.001) for PCV. HDL, LDL and APOB levels were associated with PCV (OR = 0.001, P = 0.004; OR = 0.099, P = 0.010; OR = 0.839, P = 0.018). Higher level of TC was potently associated with increased risk of PCV (OR = 109.8, P = 0.000). LIPC rs10468017 was a risk factor for PCV (OR = 11.68, P = 0.000). CETP rs3764261 conferred a decreased risk for PCV (OR = 0.08, P = 0.000). No associations of LPL rs12678919 or CETP rs173539 with PCV were found. Mean level of HDL increased with T allele of the CETP gene (p = 0.026): 1.24 mmol/L (±0.31) for the GG genotype and 1.66 mmol/L (±0.54) for the TT genotype. Additionally, T allele was associated with the following increase in APOA1: 136.78 mg/dl (±20.53) for the CC genotype and 149.57 mg/dl (±22.67) for the TT genotype of LIPC and 137.91 mg/dl (±20.36) for the GG genotype and 162.67 mg/dl (±22.50) for the TT genotype of CETP gene.

**Conclusion:**

Our study suggested that the significant association was found between hyperlipidemia, the serum levels of TC, HDL, LDL and APOB and PCV. The result of present study also showed that the association of LIPC rs10468017 and CETP rs3764261 with PCV.

## Introduction

Polypoidal choroidal vasculopathy (PCV) is an important form of maculopathy among Asian elderly [1–3], and it manifests as orange-reddish, polypoidal structures at the posterior pole [4]. Indocyanine green angiography (ICGA) is a gold standard to diagnose PCV and characterized by a network of vessels with two distinct components: a complex of branching vessels and multiple, terminal, polypoidal lesions [5–8].

Previous studies showed strong associations between the lipid metabolism associated genes including the hepatic lipase (LIPC) locus rs10468017, lipoprotein lipase (LPL) locus rs10468017 and cholesterol ester transfer protein (CETP) based on the drusen that is a mound between the RPE cell and its basement membrane and mainly consist of liquids and the development of age-related macular disease (AMD) [9–11]. These discoveries in the lipid pathway provided new insight into the pathogenesis of AMD, although other studies have found no association with AMD [12,13]. However, there are limited and inconsistent results evaluating the association between the lipid-associated genes and the development of PCV.

A change of plasma lipid/lipoproteins concentration is considered to be one of the risk factors of PCV development. A clinical trial suggests lower low-density cholesterol (LDL), is a protective factor for PCV, whereas higher high-density lipoprotein (HDL) levels and an increased risk of PCV [10]. In contrast, an epidemiological study report that higher HDL level is associated with a lower risk of PCV and there are no evident associations between LDL or total cholesterol and PCV [13]. To date, there are limited data to analyze apolipoprotein concentrations and epidemiological results of association between serum lipid biomarkers and PCV are inconsistent. Therefore, it is necessary to acquire more evidence to distinguish that the role of lipid/lipoproteins concentrations in the development of PCV.

What’s more, although several studies reported that drusen was frequently seen in PCV eyes and it is a vascularized complication [14], there are limited reports comprehensively evaluating the association of the lipid metabolism and genetic variants with the development of PCV, especially in Asian population.

Thus, we performed this study to comprehensively analyze the role of gene variations in the HDL cholesterol metabolic pathway and firstly delineate the systemic risk factors, serum levels including high-density lipoprotein (HDL), low density lipoprotein (LDL), total cholesterol (TC), triglycerides (TG), apolipoprotein A1 (APOA1) and apolipoprotein B (APOB) and genetic variants in the HDL metabolic pathway in cases and controls to further evaluate the lipid-PCV association in China population.

## Materials and methods

### Subjects

A total of 216 unrelated Chinese participants were enrolled in this case-control cohort from January 2018 to December 2018; specifically, 66 patients had PCV, and 150 individuals without age-related maculopathy (ARM) were studied as controls. The study subjects were recruited from the Department of Ophthalmology of Peking University People’s Hospital, and the study was approved by the Ethical Committee of Peking University People’s Hospital. An informed consent process was established following the guidelines of the Helsinki Declaration, and all subjects signed consent forms. All subjects received a standard ophthalmic examination, including visual acuity, slit-lamp biomicroscopy, and dilated fundus examinations, performed by two retinal specialists to avoid potential diagnosis bias. All cases with PCV underwent fluorescein angiography, optical coherence tomography (OCT), and indocyanine green angiograms with HRA2 (Heidelberg Engineering, Heidelberg, Germany). The diagnosis of PCV was based on indocyanine green angiography (ICGA) results, which showed a branching vascular network that terminated in aneurysmal enlargements. Exclusion criteria included any eye with any other macular abnormalities, such as pathological myopia, presumed ocular histoplasmosis, idiopathic choroidal neovascularization (CNV), and other secondary CNV (diabetic retinopathy). Normal controls were defined as having no clinical evidence of macular disease in any eyes, excluding subjects with severe cataracts to avoid choose bias. Data on hypertension, diabetes mellitus (DM), hyperlipidemia, asthma and coronary artery disease (CAD) was acquired by a questionnaire.

### Serum analysis

Fasting serum samples were collected on the same day (January 2018 to December 2018) as the ophthalmic examinations and were analyzed for TC, TG, HDL and LDL, APOA1, APOB, respectively, in the clinical laboratory at the Peking University People’s Hospital. Authors have terminal system to check patient blood examinations results.

### Genetic analysis

Blood samples were collected once participants clearly diagnosed and stored at -80°C before DNA extraction. Genomic DNA was extracted from venous blood leukocytes using a genomic extraction kit (Beijing eBios Biotechnology Co., Ltd.), and genotyping was performed by matrix-assisted laser desorption/ionization time-of-flight mass spectrometry (MALDI-TOF-MS), as previously described [15]. Briefly, approximately 30 ng of genomic DNA was used to genotype each sample. The DNA samples were amplified, and the PCR products were used for locus-specific single-base extension reactions. The resulting products were desalted and transferred to a 384 SpectroCHIP array (Sequenom, San Diego, CA, USA). Allele detection was performed using MALDI-TOF-MS. The mass spectrograms were analyzed using MassARRAY Typer software version 4.0 (Sequenom, San Diego, CA, USA). Genotyping was performed on January 2019 and authors had reports of every subject to identify individual participants during or after data collection.

### Statistical analysis

The data were analyzed using SPSS (version 16.0; SPSS Science, Chicago, IL). All SNPs were assessed for Hardye Weinberg equilibrium (HWE) using chi-square tests. Differences in systemic risk factors, alleles and genotypes frequencies were assessed using chi-squared tests and serum lipids levels were analyzed by unpaired Student’s t-tests between cases and controls. We analyzed the association between PCV and systemic risk factors, serum lipids and genetic variants (allele and genotypes frequencies) using logistic regression models to estimate the odds ratios (ORs) with 95% confidence intervals (CIs). We also determined the mean levels of serum lipids according to 4 SNPs genotype using ANOVA (post hoc Bonferroni test) and Student’s t-tests. P<0.05 were considered to be statistically significant.

## Results

Of all the participants (216 cases), 66 were PCV patients (mean age of 69.89±9.18 years, 53.0% males), and 150 were the controls (mean age of 69.49±9.00 years, 44.0% males). Demographic characteristic of the study population are shown in Table 1.Patients with PCV were more likely to suffer from hyperlipidemia (P<0.001). No association of age, gender hypertension, CAD, diabetes, and asthma with PCV was found (P>0.05). In multivariable analysis given in Table 2, the remained significant factors were hyperlipidemia (OR = 1.19, P = 0.001) and other factors were still not statistically significant.

**Table 1:** Systemic risk factors analysis between PCV and control.

	PCV	Control	P
	(n = 66)	(n = 150)	value
Age			0.761
Mean±SD	69.89±9.18	69.49±9.00	
Gender, n (%)			0.220
Male	35 (53.0)	66 (44.0)	
Female	31 (47.0)	84 (56.0)	
Hypertension, n (%)	32 (48.5)	92 (61.3)	0.079
Hyperlipidemia, n (%)	6 (9.1)	51 (34.0)	<0.001
Coronary artery	6 (9.1)	26 (17.3)	0.116
disease, n (%)			
Diabetes, n (%)	12 (18.2)	28 (18.7)	0.933
Asthma, n (%)	2 (3.0)	4 (2.7)	0.867

**Table 2:** Systemic risk factors analysis by logistic regression.

	PCV (n = 66) vs. Control (n = 150)
	P value OR (95% CI)
Age	0.86 1.00 (0.97,1.04)
Gender	0.245 0.69 (0.37,1.29)
Hypertension	0.583 0.83 (0.42,1.62)
Hyperlipidemia	0.001 1.19 (1.07,1.51)
Coronary artery disease	0.204 0.51 (0.18,1.44)
Diabetes	0.137 1.94 (0.81,4.65)
Asthma	0.755 0.75 (0.13,4.51)

The serum lipid levels of the cases and controls are provided in Table 3. In univariate analysis, patients with PCV had significantly lower levels of HDL and LDL (1.16 vs. 1.31 mmol/L, P = 0.001 and 2.72 vs. 3.11 mmol/L, P = 0.002). The APOA1 and APOB levels were significant lower in patients than the controls (P<0.001). In addition, in multivariable analysis (Table 4), HDL, LDL, APOB levels were remained to be associated with PCV (OR = 0.001, P = 0.004; OR = 0.099, P = 0.010; OR = 0.839, P = 0.018; OR = 0.000, P = 0.000) while APOA1 levels were no longer significant. Nevertheless, higher levels of TC tended to be potently associated with increased risk of PCV (OR = 109.8, P = 0.000). Other factors were not statistically significant.

**Table 3:** Levels of serum lipids analysis between PCV and controls.

	PCV	Control	P
	(n = 66)	(n = 150)	value
Age			0.761
Mean±SD	69.89±9.18	69.49±9.00	
Gender, n (%)			0.220
Male	35 (53.0)	66 (44.0)	
Female	31 (47.0)	84 (56.0)	
HDL (mm/L)	1.16±0.29	1.31±0.32	0.001
LDL (mm/L)	2.72±0.91	3.11±0.81	0.002
TC (mm/L)	4.82±1.05	5.11±0.95	0.050
TG (mm/L)	1.72±0.82	1.65±1.09	0.641
APOA1 (mg/dl)	131.24±19.36	143.06±19.36	<0.001
APOB (mg/dl)	88.39±24.66	100.73±21.13	<0.001

**Table 4:** Levels of serum lipids analysis by logistic regression.

	PCV (n = 66) vs. Control (n = 150)
	P value OR (95% CI)
Age	0.341 0.98 (0.93,1.02)
Gender	0.357 0.61 (0.20,1.76)
HDL (mm/L)	0.004 0.001 (0.000,0.106)
LDL (mm/L)	0.010 0.099 (0.017,0.569)
TC (mm/L)	0.000 109.8 (1.65, 1249.9)
TG (mm/L)	0.079 0.43 (0.171,1.10)
APOA1 (mg/dl)	0.403 0.974 (0.917,1.035)
APOB (mg/dl)	0.018 0.839 (0.839,0.984)

Next, SNPs statistics were analyzed in 66 patients with PCV compared with 150 controls except rs173539 (66 cases and 147 controls). Table 5 and Table 6 gave details of genotype and allele frequencies statistics. All 4 SNPs showed no significant deviation from HWE in the control and PCV group (P>0.05), except rs173539 in the PCV group (P<0.01). We found that the risk allele frequency of LIPC rs10468017 was significantly associated with PCV in the patients with PCV (P = 0.000, OR = 11.68). However, the frequency of the minor allele of CETP rs3764261 tended to be a protective role (P = 0.000, OR = 0.08). CETP rs173539 and LPL rs12678919 did not show significant associations with the development of PCV (P>0.05). Interestingly, The homozygosity of risk alleles (Genotype) for 4 SNPs conferred no association of PCV (P>0.05).

**Table 5:** Allele frequency associations analysis.

Gene	SNP	Allele	PCV	Control	P^a^ Value	P^b^ Value	OR (95%CI)
		Risk allele					
LIPC	rs10468017	T	28 65		0.000	0.000	11.68(4.16,32.82)
CETP	rs3764261	T	27 48		0.485	0.000	0.08(0.03,0.25)
	rs173539	T	4 12		0.850	0.599	0.73(0.23,2.32)
LPL	rs12678919	G	8 21		0.901	0.356	1.61(0.58,4.45)

P^a^. chi-square test

P^b^. logistic regression test

**Table 6:** Genotype frequency associations analysis.

Gene	SNP	Genotype			PCV	Control	P^a^ Value	P^b^ Value	OR (95%CI)
LIPC	rs10468017	CC	CT	TT	41/22/3 89/57/4		0.660	0.982	1.00(0.58,1.76)
LPL	rs12678919	AA	AG	GG	58/8/0 129 /21/0		0.000	0.721	0.84(0.33,2.14)
CETP	rs173539	CC	CT	TT	64/0/2 141/0/6		1.000	0.710	0.86(0.38,1.93)
	rs3764261	GG	GT	TT	40/25/1 104/44/2		0.453	0.832	0.77(0.07,8.72)

P^a^. chi-square test

P^b^. logistic regression test

Finally, we explored the mean levels of serum lipids according to LIPC genotype (CC, CT, TT) and CETP genotype (GG, GT, TT). As seen in Table 7, mean level of HDL increased with each T allele of the CETP gene (p = 0.026). Mean level of HDL was 1.24 mm/L (±0.31) for the GG genotype and 1.66 mg/L (±0.54) for the TT genotype. Additionally, mean level of APOA1 increased with each T allele of the LIPC gene (Mean level of APOA1 was 136.78 mg/dl (±20.53) for the CC genotype and 149.57 mg/dl (±22.67) for the TT genotype) and CETP gene (137.91 mg/dl (±20.36) for the GG genotype and 162.67 mg/dl (±22.50) for the TT genotype). LDL, total cholesterol triglycerides and APOB were not significantly associated with LIPC and CETP.

**Table 7:** Associations of serum lipids with LIPC and CETP genotypes.

	LIPC rs10468017				CETP rs3764261			
	CC	CT	TT	P	GG	GT	TT	P
				value				value
HDL (mm/L)	1.23±0.31	1.31±0.33	1.33±0. 36	0.134	1.24±0.31	1.30±0.32	1.66±0.54	0.026
LDL (mm/L)	3.01±0.88	2.99±0.82	2.52±0.93	0.341	2.97±0.89	3.02±0.80	2.97±0.60	0.908
TC (mm/L)	4.98±0.99	5.11±0.96	4.80±1.15	0.545	4.98±0.98	5.09±1.03	5.29±0.45	0.629
TG (mm/L)	1.62±0.82	1.75±1.28	1.69±0.91	0.660	1.61±0.95	1.81±1.14	1.36±0.46	0.351
APOA1 (mg/dl)	136.78±20.53	142.94±18.44	149.57±22.67	0.031	137.91±20.36	141.65±18.81	162.67±22.50	0.034
APOB (mg/dl)	96.35±23.08	98.60±22.87	89.71±22.17	0.552	96.47±23.15	97.99±22.99	96.33±12.43	0.902

## Discussion

In our study, a significant association was found between hyperlipidemia and PCV, whereas other factors including age, gender, hypertension, CAD, diabetes and asthma were not associated with PCV. We found that hyperlipidemia conferred an increased risk for PCV (OR = 1.19, P = 0.001), which is consistent with our previous published data [16]. However, there was result that no significant difference in hyperlipidemia between PCV and AMD [16]. Previous literature revealed that males are prone to suffer PCV than females [17,18,19]. Our study showed the result of all the 66 cases, 35 participants were male PCV patients (53.0%) and 66 were the male controls (44.0%). Gender was not significantly associated with PCV. We consider that this discrepancy could result from sample size of ethnic group. Other that, the associations between hypertension, CAD, DM, asthma and PCV have been inconsistent [20,21]. Interestingly, PCV is featured by the formation of a branching vascular network (BVN) and polypoidal dilated lesions at vessel termini [22]. The spontaneous rupture of these polypoidal dilations may result in subretinal hemorrhages (SRH) and an abrupt decrease in the visual acuity. We speculated that blood pressure is associated with the formation of polypoidal dilated lesions and vascular rupture. Many study results in agreement with this hypothesis, especially recurrent SRH [23,24]. However, our research has come to the opposite conclusion and previous study showed hypertension has no significant association with the development of PCV [25]. Thus, the role of hypertension played in the pathogenesis of PCV merit further investigations through larger randomized studies. Compared with studies on AMD, there are fewer researches on risk factors of PCV that mainly has a high prevalence in Asian countries. Thus, a multi-center study needs to be further performed to confirm the role of gender and other systemic risk factors in the development of PCV. We found that CAD, DM and asthma conferred no risk for PCV whereas other data showed different results [10,17]. Hyperlipidemia is a known risk factor for CAD and it increases the incidence of PCV according to our results, which reminds that the ethnicity sample size needs to be expanded for further research. There is little research about asthma and the role of asthma remains to be determined.

There are growing data to study the participation of lipid metabolism in AMD pathogenesis. They evaluated that photoreceptors supplied RPE with an influx of lipids to generate ATP and damaged mitochondria lose the capacity to generate ATP [26]. It is warranted to collect more evidences to reflect the relationship between the level of serum lipids and the PCV. We found that higher serum level of HDL and LDL were protective factors of PCV and higher level of LDL was negatively associated with PCV. Many studies that investigated the association between PCV and HDL have been not consistent. Higher HDL levels reported to be positively associated in some studies while others reported decreased PCV risk [10,13]. Likewise, the strength of such relevance between LDL levels and PCV is also widely variable. Chen et al reported serum levels of LDL did not reach statistical significance in patients with PCV compared to the controls [13]. The results data on the association of HDL and LDL are controversial. Generally, HDL is helpful the prevention of atherosclerosis and coronary heart disease. LDL is a carrier of cholesterol, which is transported to tissues, and HDL is a convert. High LDL directly promotes atherosclerosis. We speculated that the function difference of lipids in retina and it is warranted to conduct a larger sample size research to analyze the point. Additionally, we identified that the serum TC levels was a strongly risk factor. However, no significant differences were found for TC levels between the cases and controls. It is crucial to perform a larger sample size study to further confirm that the role of serum lipid levels in the development of PCV. Further, we found that the concentrations of APOA1 and APOB are parallel to HDL and LDL and the level of APOB play a protective role in PCV, which is inconsistent with previous studies [27]. PCV as a special type of AMD from the previous point whereas this statement should be controversial because it does not fully coincide with AMD and further research is needed. We speculated that this discrepancy might be due to different sample sizes. In a recent study [28], APOE and CETP genotype influenced HDL and APOA1 levels and both were significantly associated with AMD. We did take genetic polymorphism into account. LIPC, a critical enzyme, as hepatic triglyceride lipase involved in HDL metabolism [29]. In our analysis, the LIPC rs10468017 was associated with PCV (OR = 11.68) and level of APOA1 is increasing with allele T (P = 0.031), which is inconsistent with recent studies [13, 17, 25, 30]. That the effect of APOA1 was found by genotype classification instead of regression analysis indicated that further study is focused on genes associated with lipid metabolism.

Besides, CETP facilitates the reverse cholesterol transport by mediating the transfer of additional lipids from HDL to LDL in the systemic circulation [31]. In retina, CETP has the capacity to promote the maturation of HDL by transferring the oxidized lipids from the outer segments of the photoreceptors to retina pigment epithelial cells then excreted back into the system circulation [32, 33]. CETP SNP rs3764261 (the allele T) was confirmed to be significantly negatively associated with PCV whereas an association of rs173539 in CETP with PCV was not observed, which was not in consistent with many results. There were three studies indicated that CETP rs3764261 meaningfully increased the risk for PCV [13,34,35]. Further, that the apparent high levels of APOA1 and HDL are associated with T allele of CETP rs3764261 supply evidence that CETP rs3764261 play a protective role in PCV. Other else, we did not find any association of LPL rs12678919 and CETP rs173539 in allele and genotype frequency with PCV. The strength of this gene-analysis may be limited in that we just included analysis of participants in whom genetic data was available due to funding constraints. These results could provide grounds for further forward cohort research and make clinical doctors to pay attention to the risk factors exposure and evaluate the prognosis of PCV patients.

In conclusion, our study suggested that the significant associations were found between hyperlipidemia, the serum levels of TC, HDL, LDL and APOB and PCV. In addition, the LIPC rs10468017 conferred an increased risk for PCV and the CETP rs3764261 play a protective role in PCV. Serum lipids level is associated with the genotype and further research is warranted.

## Supporting information

S1 Table All measured data shown in the S1 Table. (XLSX)
